# Transcriptome Characterization of Repressed Embryonic Myogenesis Due to Maternal Calorie Restriction

**DOI:** 10.3389/fcell.2020.00527

**Published:** 2020-06-26

**Authors:** Jun He, Ying He, Bing Yu, Xuelian Wang, Daiwen Chen

**Affiliations:** ^1^Institute of Animal Nutrition, Sichuan Agricultural University, Chengdu, China; ^2^Key Laboratory of Animal Disease-Resistance Nutrition, Ministry of Education, Chengdu, China; ^3^ABlife Inc., Wuhan, China

**Keywords:** embryonic myogenesis, development, transcriptome, myofiber, nutrition

## Abstract

Fetal malnutrition decreases skeletal myofiber number and muscle mass in neonatal mammals, which increases the risk of developing obesity and diabetes in adult life. However, the associated molecular mechanisms remain unclear. Here, we investigated how the nutrient (calorie) availability affects embryonic myogenesis using a porcine model. Sows were given a normal or calorie restricted diet, following which skeletal muscle was harvested from the fetuses at 35, 55, and 90 days of gestation (dg) and used for histochemical analysis and high-throughput sequencing. We observed abrupt repression of primary myofiber formation following maternal calorie restriction (MCR). Transcriptome profiling of prenatal muscles revealed that critical genes and muscle-specific miRNAs associated with increased proliferation and myoblast differentiation were downregulated during MCR-induced repression of myogenesis. Moreover, we identified several novel miRNA-mRNA interactions through an integrative analysis of their expression profiles, devising a putative molecular network involved in the regulation of myogenesis. Interestingly, NC_010454.3_1179 was identified as a novel myogenic miRNA that can base-pair with sequences in the 3′-UTR of myogenic differentiation protein 1 (MyoD1). And we found that this UTR inhibited the expression of a linked reporter gene encoding a key myogenic regulatory factor, resulting in suppression of myogenesis. Our results greatly increase our understanding of the mechanisms underlying the nutrient-modulated myogenesis, and may also serve as a valuable resource for further investigation of fundamental developmental processes or assist in rational target selection ameliorating repressed myogenesis under fetal malnutrition.

## Introduction

Epidemiological studies have indicated that being small for gestational age (SGA) not only leads to increased prenatal and neonatal mortality, but also increases the risk of developing obesity, coronary heart disease, hypertension, and non-insulin-dependent diabetes in adult life (Valdez et al., 1994). Failure of the fetus to achieve its optimal growth potential can have many causes, however, the main reasons involve poor fetal nutrition or lack of adequate oxygen supply to the fetus (Tuuli et al., 2011). Although nearly every fetal organ is affected by being SGA, skeletal muscle is particularly at risk because blood flow and nutrient supply are preferentially allocated to support the growth of vital organs under conditions of fetal malnutrition (Beltrand et al., 2008).

Skeletal muscle development is a highly-ordered process involving a series of separable events including the myogenic progenitor cell determination and proliferation, myoblast differentiation, and subsequent myotube modulation. Suppressed fetal skeletal muscle growth cannot generally be fully compensated for after birth, as individuals who are born with low birth weight usually have reduced muscle mass in adulthood (Yliharsila et al., 2007). Muscle mass is mainly determined by the number and size of myofibers. However, the myofiber number is set at the time of birth and disruption of the myofiber formation cannot be fully rescued (Widdowson et al., 1972). Importantly, increasing evidences has shown that suppressed myogenesis in human fetuses may be a major contributing factor to the increased risk of sacropenia, obesity, and diabetes in later life (Srikanthan and Karlamangla, 2011). The importance of muscle mass and function in numerous diseases underpins the necessity of understanding how the prenatal myogenesis is regulated.

The pig (*Sus scrofa*) is an excellent model species for use in biomedical research as it is closely related to humans in terms of anatomy, genetics, and physiology. Importantly, both species are omnivorous and their organs generally have common functional features. In pigs, the establishment of myofibers involves two major waves of fiber generation, i.e., a primary fiber generation occurring from 35 until 55 days of gestation (dg), followed by a second generation which forms between 55 and 90–95 dg (Wigmore and Stickland, 1983). Studies on pigs and other animal species have shown that fetal nutrition plays a critical role in establishing myofiber number, and insufficient nutrient supply during gestation significantly reduces myofiber number in neonatal animals (Dwyer et al., 1994). Moreover, insufficient fetal nutrient supply can also affect the fiber type composition, generally favoring decreased type II fiber expression (Zhu et al., 2004). However, the mechanisms involved in the restriction of myofiber number and modulation of fiber type resulting from fetal undernutrition are not well understood.

To explore the patterns of embryonic myogenesis and how fetal skeletal muscle growth adapts to nutrient availability, we performed a transcriptome analysis of prenatal skeletal muscles using high-throughput sequencing (Chu and Corey, 2012). We generated skeletal muscle transcriptome profiles for Large White pigs at critical prenatal stages, and used an integrative analysis of miRNA-mRNA profiles to identify several novel miRNA-mRNA interactions. The results highlight the importance of using integrative methods to identify regulated miRNAs and their targets, and contribute to our understanding of the molecular mechanisms underlying the repression of myogenesis associated with fetal malnutrition.

## Results

### MCR Inhibited Primary Skeletal Myofiber Formation During Embryonic Myogenesis

**FIGURE 1:**
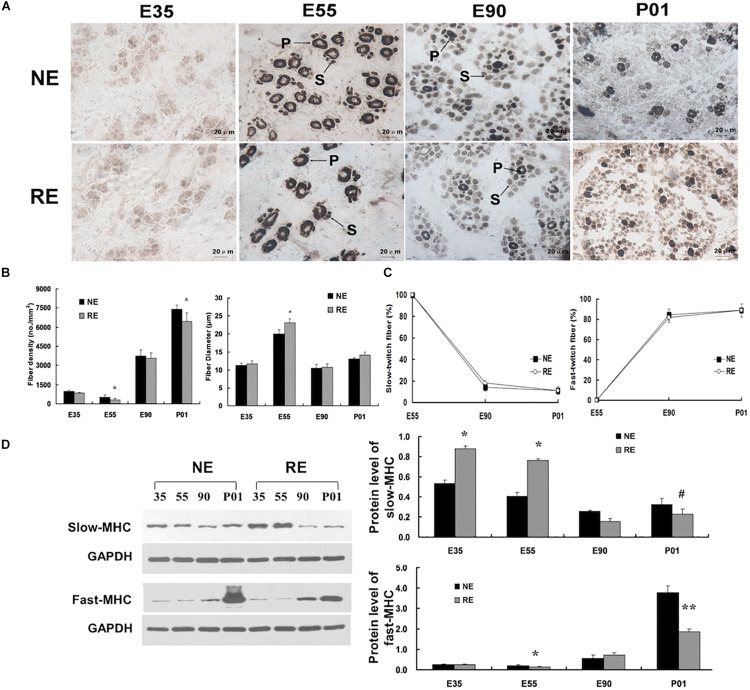
Morphology and Myofiber distribution of the *longissimus* muscles. **(A)** Myofiber morphology of *longissimus* muscles at different developmental stages. Sections were stained by using ATPase staining method. All areas were photographed at a magnification of ×400; NE/RE indicate normal/reduced calorie supply during gestation; E35, E55, and E90 indicate samples collected at 35, 55, and 90 days of gestation (dg), and P01 indicate 1 day post-partum; P, primary fibers; S, secondary fibers. **(B)** Myofiber density and size. **(C)** Ratio of total slow-/fast-twitch fibers at distinct stages. **(D)** Protein levels of slow-/fast-twitch myosin heavy chain (MHC). *P < 0.05, **P < 0.01, ^#^0.05 < *P* < 0.10.

The number of pigs born per litter was not affected by maternal calorie restriction (MCR). However, MCR decreased fetus size in the late gestation period and reduced the birth weight (Supplementary Table S1). To explore the distribution of muscle fibers, a conventional histochemical method based on the sensitivity of actomyosin ATPase to acidic pH (pH 4.35) was used (Brooke and Kaiser, 1970). Sections of the *longissimus* muscles at 35 dg showed typical primary fiber characteristics of Figure 1A. By 55 dg, the primary fibers had increased in size and the secondary fibers had formed on the surface of primary fibers, which is consistent with previous reports that the secondary fibers form at 50∼55 dg (Wigmore and Stickland, 1983). At this stage, the primary fibers comprised the majority of myofibers, however, MCR significantly reduced primary fiber density (Figures 1A,B and Supplementary Figure S1). After 55 dg, the fibers had increased in number but reduced in size, and MCR decreased its density in neonatal pigs. The repressed myofiber formation and reduced birth weight reflect the MCR-induced suppression of prenatal myogenesis.

The fiber type composition was determined by an actomyosin ATPase-based method (Brooke and Kaiser, 1970). The fiber types could not be distinguished at 35 dg, as the ATPase activity was not detected at this developmental stage (Figure 1A). At 55 dg, the ATPase activity was mainly present around the periphery of the primitive myotubes and the central area was devoid of myofibrils, indicating that the ontogenesis of type I fibers occurred before that of type II fibers. However, after 55 dg, the percentage of type II fibers has increased and comprised the majority of total myofiber number (Figure 1C). Interestingly, nearly all the primitive myotubes still exhibited central vacuolation at 90 dg under calorie restriction, suggesting that MCR delayed the maturation of skeletal myotubes. Moreover, MCR led to elevated slow-MHC protein levels at 35 and 55 dg. Fast-MHC protein levels were presented in low amounts at the early stage, but increased markedly after 55 dg. Importantly, MCR significantly reduced the protein levels of fast-MHC in neonatal pigs (Figure 1D).

### Transcriptome Profiles Revealed a Critical Developmental Transition During Embryonic Myogenesis

**FIGURE 2:**
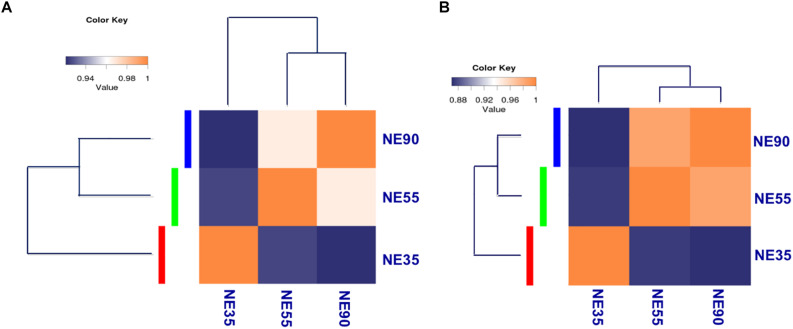
Transcriptome profiling reveals distinct developmental stages during embryonic myogenesis. Sample correlation matrix indicates a sharp transition of gene expression pattern **(A)** and miRNA expression pattern **(B)** between 35 and 55 dg. NE35, NE55, and NE90 indicate samples collected from NE group at 35, 55, and 90 dg, respectively. The correlation of gene expression patterns between distinct stages was analyzed by using cor in R package, and correlation matrix was plotted based on Pearson correlation coefficients between paired samples.

For transcriptome sequencing, 26.69 million raw reads were generated, and 21.78 million clean reads remained after filtering. Most reads were mapped to coding regions, indicating that the data were highly reliable (Supplementary File S1). Gene expression levels were calculated and normalized to reads per kilobase of exon model per million mapped reads (RPKM), and differentially expressed genes (DEGs) were identified by using edgeR (Mortazavi et al., 2008; Robinson et al., 2010). Correlations between gene expression patterns for distinct stages were analyzed using the *cor* function in the R package, and a correlation matrix was plotted based on Pearson’s correlation coefficients between paired samples. As shown in Figure 2A, the samples from 55 and 90 dg (NE55 and NE90) were initially clustered together, suggesting that their expression patterns were similar (*R*^2^ = 0.934). In contrast, the NE35 transcription profile differed considerably from those of NE55 and NE90, indicating that a critical developmental transition occurred between 35 and 55 dg. More DEGs were identified at the early transition period (NE35/NE55) than in that from 55 to 90 dg. We analyzed the functional categories of the DEGs using DAVID^1^, and found that the DEGs identified at the early transition period were highly enriched in myogenic processes such as the muscle organ development (6.27%, *P* < 0.001), muscle contraction (8.27%, *P* < 0.01), and fiber formation (9.52%, *P* < 0.01). Meanwhile, there was a tendency for more DEGs involved in biopolymer metabolism between 55 and 90 dg (Supplementary File S2).

For small RNA-sequencing, 6.4 million reads per sample were generated by RNA sequencing, approximately 96% of which were clean reads. After eliminating adaptor and low-quality reads, most clean reads could be mapped to the pig reference genome (Supplementary File S1). Based on miRDeep2 analysis, we identified 415 miRNAs, including 281 known miRNAs and 134 candidate novel miRNAs, at levels of at least 10 reads in at least one of the samples (Friedlander et al., 2012; Kozomara and Griffiths-Jones, 2014). Differentially expressed miRNAs were identified through the Bioconductor package edgeR (Robinson et al., 2010). Interestingly, the correlation matrix showed that the NE35 miRNA expression pattern differed considerably from those of NE55 and NE90 (Figure 2B), which agreed well with the mRNA expression patterns observed during the prenatal myogenesis. Both indicated the occurrence of a critical developmental transition between 35 and 55 dg.

### MCR Represses the Developmental Transition in Early Gestation

**FIGURE 3:**
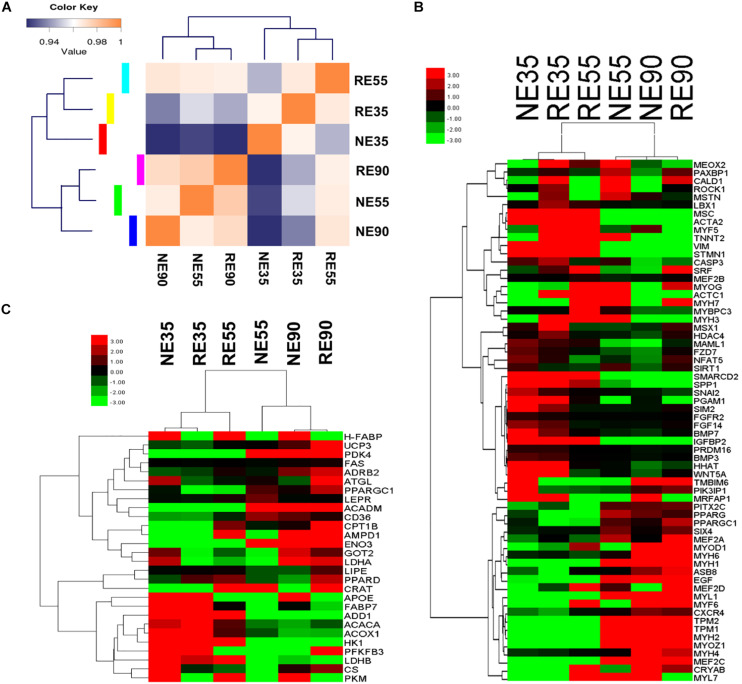
Whole genome expression characterization of the effect of MCR on muscle development. **(A)** Sample correlation matrix indicates the influence of MCR on gene expression patterns. **(B)** Influence of MCR on expression profiling of myofiber development genes. **(C)** Influence of MCR on expression profiling of metabolic type-related genes. NE35, NE55, and NE90 indicate samples collected from NE group at 35, 55, and 90 dg, respectively, RE35, RE55, and RE90 indicate samples collected from RE group at 35, 55, and 90 dg, respectively.

A total of 644 genes were found to be significantly modulated by MCR at 35 dg. In contrast, only 332 and 252 DEGs (NE vs. RE) were identified at 55 and 90 dg, respectively (Supplementary File S3). Most DEGs were enriched in biological pathways relating to cellular events, organ development, and nutrient metabolism. Interestingly, the RE35 gene expression pattern was similar to that of RE55 (Figure 3A); as a critical developmental transition was observed between 35 and 55 dg under normal conditions, this result suggested that myogenesis was suppressed as a result of MCR. The influences of MCR on the transcript levels of genes involved in muscle development and metabolism were depicted in heatmaps (Figures 3B,C), and critical genes were validated by qPCR (Supplementary File S4). For most genes, the qPCR results agreed well with the sequencing data, which further suggested that the sequencing data were highly reliable. We showed that the expression levels of genes involved in the regulation of progenitor cell migration and proliferation (i.e., *SPP1*, *SIRT1*, *BMP7*, and *PPARG*) and embryonic organ development (i.e., *PIK3IP1*, *IGFBP2*, and *EGF*) peaked at 35 dg, however, at this stage, these genes were down-regulated by MCR. In contrast, the expression levels of *MSTN* and *HDAC4*, negative regulators of myogenesis, were upregulated due to MCR at 35 dg. This may have contributed to repressed primary myofiber formation under conditions of MCR. At 55 dg, the expression levels of *VIM*, *STMN1*, and *IGFBP2* were increased following MCR, whereas those of *TPM1*, *TPM2*, *NFAT5*, and *EGF* were downregulated. Interestingly, MCR led to up-regulation of some myogenesis-related genes at 90 dg (*NFAT5*, *MAML1*, and *SIRT1*). Two genes involved in energy metabolism, *UCP3* and *PDK4*, were expressed in low amounts at 35 dg, but the expression of both genes was upregulated after this stage. Moreover, two critical glycolytic genes (*HK1* and *LDHB*) were downregulated with MCR at 35 dg, whereas genes with roles in lipid utilization (*LIPE* and *FABP7*) were upregulated at this stage.

**FIGURE 4:**
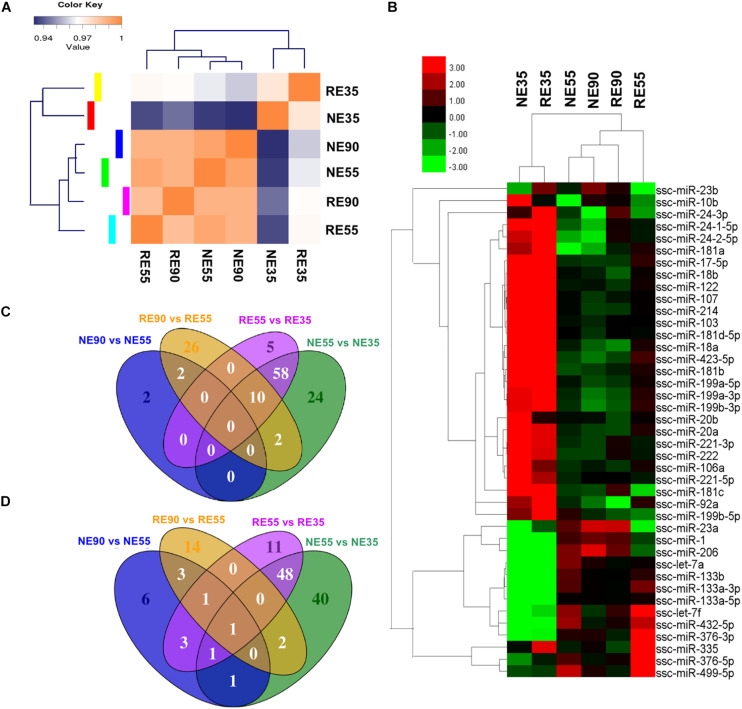
Nutrient-regulated miRNA expression pattern during prenatal muscle development. **(A)** Sample correlation matrix indicates a critical role of MCR in miRNA expression. **(B)** Heatmap shows the influence of MCR on expression profiling of muscle-specific miRNAs. **(C,D)** Venn diagrams show the overlap among DE miRNAs identified in various comparisons [**(C)** shows the upregulated miRNAs, **(D)** shows the downregulated miRNAs]. NE35, NE55, and NE90 indicate samples collected from NE group at 35, 55, and 90 dg, respectively, RE35, RE55, and RE90 indicate samples collected from RE group at 35, 55, and 90 dg,

A greater number of differentially expressed miRNAs (NE vs. RE) were identified at 35 dg than at 55 or 90 dg (Supplementary File S5). A correlation matrix showed that the miRNA expression pattern differed between the NE and RE groups (Figure 4A). The expression profiles of muscle-specific miRNAs are shown in Figure 4B and Supplementary File S4. We found that the expression levels of several muscle-specific miRNAs (miR-10b, miR-106a, miR-221-5p, and miR-222) peaked at 35 dg, however, the expression levels of these miRNAs were downregulated by MCR at this early stage. The expression levels of miR-133a and miR-499-5 peaked at 55 dg, whereas those of miR-1, miR-23a, and miR-206 peaked at 90 dg. Interestingly, the expression levels of miR-1, miR-23a, and miR-206 were decreased with MCR at 55 dg. Both the miR-376 and miR-499 were highly expressed at 55 dg, and MCR further increased the expression levels of both miRNAs at this stage. The DE muscle-specific miRNAs identified in our study offer a valuable point of reference for investigating other functional miRNAs during prenatal muscle development.

The expression profiles of all DE miRNAs were depicted in a heatmap (Supplementary Figure S2). The expression patterns of miRNAs such as the miR-424-5p, NC_010447.4_14168, and NC_010448.3_14733 were similar to that of miR-221-5p, a muscle-specific miRNA that has been shown to promote cell cycle progression and proliferation (Liu et al., 2009). These miRNAs were highly expressed at 35 dg, however, their expression were decreased at this stage following MCR. The expression patterns of miR-362, miR-542-5p, and NW_003539007.1_21524 were similar to those of miR-133 family members and their expression levels peaked at 55 dg. Additionally, the expression patterns of miR-338 and miR-378b-3p were similar to that of miR-206, suggesting that they may have a similar role in the regulation of myoblast differentiation (Chen et al., 2006; Tauli et al., 2009). Venn diagrams displaying the overlap among the DE miRNAs identified are presented in Figures 4C,D. Interestingly, 106 DE miRNAs showing differential expression between NE55 and NE35 overlapped with those differentially expressed between RE55 and RE35, suggesting that their expression is more likely to be time-dependent. Some muscle-specific miRNAs (miR-133a, miR-206, miR-221, and miR-499) were included in this category. Moreover, 64 DE miRNAs were only identified between NE35 and NE55, whereas 16 DE miRNAs were only identified between RE35 and RE55, suggesting that their expression is more sensitive to nutrient availability at the early stage. Complete list of DE miRNAs for various comparisons is provided in Supplementary File S6.

### Nutrient-Modulated Regulatory Network Controlling Embryonic Myogenesis

**FIGURE 5:**
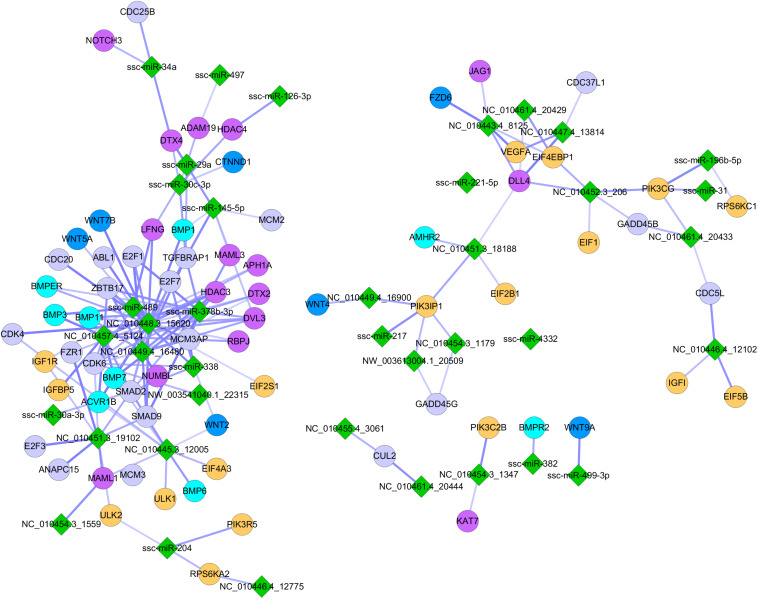
Influence of MCR on critical signaling pathways involved in embryonic myogenesis. Green diamonds indicate DE miRNAs identified between NE and RE group; Dots with different colors indicate miRNA targeted genes involving in different myogenic signaling pathways (gray: MAPK signaling pathway; yellow: mTOR signaling pathway; purple red: Notch signaling pathway; navy blue: WNT signaling pathway; wathet blue: BMP signaling pathway).

To further explore the mechanisms underlying the MCR-induced repression of myogenesis, we performed an integrated analysis of the miRNA and mRNA expression profiles. The targets of DE miRNAs (NE vs. RE) were identified based on sequence complementarity and free energy of the predicated RNA duplex (Kertesz et al., 2007). The prediction was further refined by analyzing the negative regulatory relationship between miRNAs and target mRNAs, and a non-parametric index indicating statistical coefficient for a small number of measures was used to estimate the degree of anticorrelation between putative miRNA-mRNA pairs (Wang and Li, 2009; Xin et al., 2009). The filtering criterion was set at a correlation coefficient < −0.58 and a *P* < 0.05. A total of 6,139 miRNA-mRNA regulatory pairs comprising 65 miRNAs and 2,505 mRNAs were identified and found to be modulated following MCR (Supplementary File S7). In addition to the known miRNA-mRNA pairs, some novel regulatory pairs with roles in signaling pathways critical for myogenesis (i.e., BMP, WNT, mTOR, MAPK, and Notch signaling pathways) were identified (Figure 5). These results offer an overview of the global changes occurring in the myogenic regulatory network in response to MCR.

**FIGURE 6:**
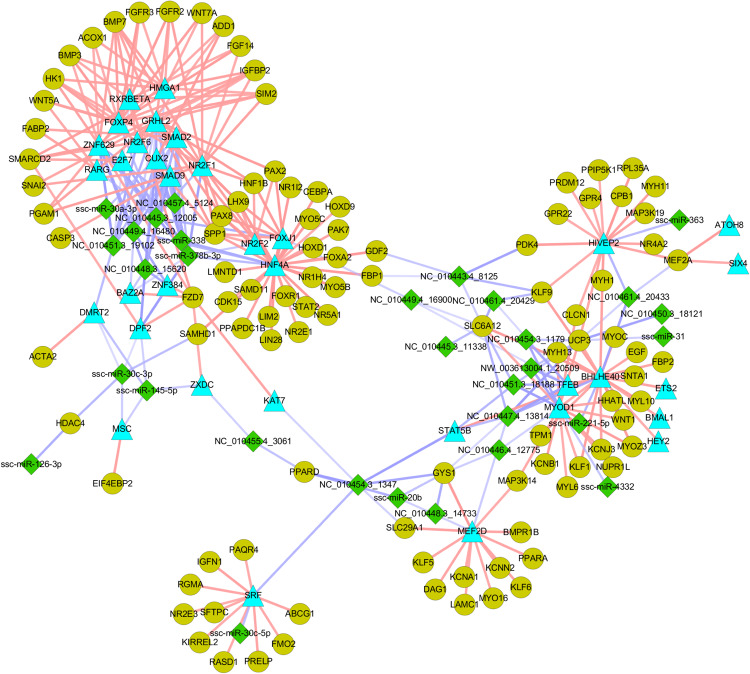
The nutrient-modulated miRNA-TF-mRNA regulatory loop associated with skeletal muscle development. Green diamonds indicate DE miRNAs identified between NE and RE group; Blue triangles indicate miRNA targeted TFs that are potentially involved in myogenesis; gray-green dots indicate potential target of the TFs that are involved in myogenesis. The miRNA-mRNA regulatory pairs were identified by using sequence-based target prediction and negative correlation of miRNA-mRNA profiles, and the targets of TFs were identified based on their regulation relationships as described in “Materials and Methods” section.

A recent study indicated that miRNAs can be “fine-tuners” of mRNA expression, leading to weak individual associations between miRNA and mRNA expression (Van Iterson et al., 2013). In this study, we introduced a “miRNA-TF-mRNA” regulatory loop, which increased the number of biologically relevant miRNA targets. Through searching the Animal Transcription Factor DataBase (TFDB)^2^, 101 TFs, including two critical for the regulation of myogenesis (MYOD1 and MEF2A), were identified among the 2,505 DE mRNAs. These TFs are targeted by 38 miRNAs (Supplementary File S8). The targets of the TFs were identified based on their coexpression relationships measured by Pearson’s correlation using the R package. Criteria for selection were a correlation coefficient > 0.90 and *P* < 0.01. More than 20,000 TF-mRNA pairs containing 49 TFs and 4,097 mRNA targets were identified (Supplementary File S9), suggesting that a complicated and cross-linked regulatory network exists during embryonic myogenesis. As shown in Figure 6, miR-338 and miR-378-3p stood out amongst the selected miRNAs, as their expression patterns were similar to those of muscle-specific miRNAs (i.e., miR-1 and miR-206) and were indicated to regulate the expression of several myogenic genes. Moreover, some novel miRNAs such as NC_010454.3_1179 and NC_010448.3_15620 were also identified as regulating the expression of several myogenic genes, implying that they are also critical for prenatal muscle development.

### NC_010454.3_1179 Is a Novel miRNA Involving in the Regulation of Myogenesis

**FIGURE 7:**
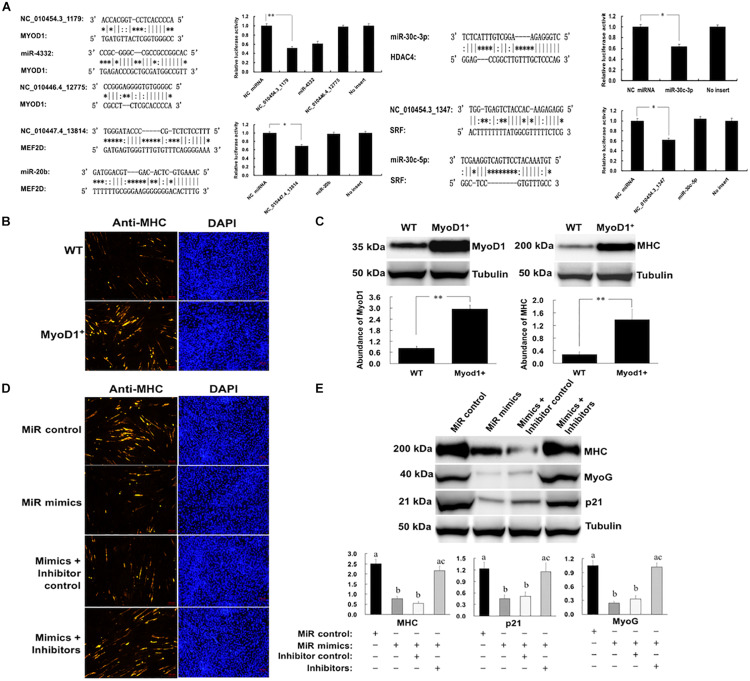
Regulation of porcine myogenesis by NC_010454.3_1179. **(A)** Validation of critical novel miRNA-mRNA regulatory pairs by luciferase assays. HEK293 cells were transfected with miRNA mimics and plasmids carrying the 3′UTR of target genes. NC: negative control miRNA (^∗^*P* < 0.05, ^∗∗^*P* < 0.01); **(B)** Effect of MyoD1 overexpression on myogenesis. The porcine myoblasts cultured in growth medium were electroporated with plasmid pcDNA.3.1 (WT) or pcDNA.3.1-MyoD1 (MyoD1^+^). Cells were continuously cultured in growth medium for 24 h after transfection and then transferred to differentiation medium for 36 h before immunostaining for MHC. **(C)** Shows the western blot analysis of the MyoD1 and MHC in the WT and MyoD1^+^ cells (^∗^*P* < 0.05, ^∗∗^*P* < 0.01). **(D,E)** Cells were electroporated with miRNA mimics, miRNA inhibitors, or their controls. Cells were cultured in growth medium for 24 h after transfection and then transferred into differentiation medium for 12 before western blot analysis of p21 and MyoG, or 36 h before western blot analysis for MHC (^a,b^Means in a same column with different letters differ significantly, *P* < 0.05).

Some critical miRNA-mRNA interactions were validated through luciferase reporter assays. MYOD1 is the master regulator of myoblast differentiation. We showed that the *MYOD1* can be a direct target of NC_010454.3_1179, as transfection with miRNA mimics resulted in significant reductions in relative luciferase activity for *MYOD1*-expressing plasmids when compared with negative control miRNA (random miRNA sequence) and the no-insert control (Figure 7A). Another critical myogenic factor, MEF2D, which was previously reported to be targeted by miR-218 (Cao et al., 2015), was a direct target of NC_010447.4_13814. While SRF and HDAC4 were shown to be targets of miR-30c-3p and NC_010454.3_1347, respectively.

The biological functions of the NC_010454.3_1179 in skeletal muscle development were further investigated, as *MYOD1* plays a vital role in myogenesis. We first validated the role of MYOD1 in the regulation of myogenesis. The *MYOD1* gene (containing the 3′-UTR) was overexpressed in porcine myoblasts and its expression validated by western blot. As shown in Figures 7B,C, overexpression of *MYOD1* in myoblasts significantly increased the levels of MYOD1 protein in these cells, resulting in enhanced myogenesis, as indicated by an increase in myosin heavy chain (MHC) expression. We then transfected myoblasts with NC_010454.3_1179 mimics and observed a significant reduction in the expression of the myogenic marker genes *MHC* and *MYOG* (Figures 7D,E). The expression of p21, a major MYOD1 target that plays a critical role in cell cycle regulation, was also downregulated after transfection of this miRNA. We showed that the NC_010454.3_1179-mediated inhibition of myogenesis was specific, because transfection the cells with a control miRNA showed no effect. In addition, specific miRNA inhibitors introduced into the cells abolished the ability of NC_010454.3_1179 to suppress the expression of myogenesis-related genes (Figures 7D,E). Together with the luciferase assays, these results suggest that the NC_010454.3_1179 is a novel miRNA that regulates myogenesis by targeting *MYOD1*.

## Discussion

To the best of our knowledge, this is the first report showing the transcriptome dynamics of the porcine prenatal muscles and how prenatal myogenesis adapts to nutrient availability. Very high-quality high-throughput sequencing data was obtained, and the expression patterns of a number of selected genes and miRNAs identified by qPCR and RNA-Seq agreed well. This suggested that our sequencing data was reliable and could be utilized for a comprehensive analysis of gene expression profiles. In pigs, postnatal muscle growth is mainly determined by the total number of myofibers, which is further determined by two major waves of fiber generation, i.e., a primary generation from 35 to approximately 55 dg, and a secondary generation from about 55 to 90 dg (Wigmore and Stickland, 1983). This indicates that the 35, 55, and 90 dg time points are critical for embryonic myogenesis. The mRNA-miRNA transcription profiles at 35 dg differed considerably from those at 55 and 90 dg, indicating that significant differences exist between primary and secondary myofiber formation in pigs.

Primary myotube formation is a complex biological process that is regulated by numerous genes and pathways. We found that several protein-coding genes involved in myofiber formation were expressed in a time-dependent manner, acting sequentially in myogenic differentiation or modulation. The regulators of muscle precursor cell proliferation, SPP1 and SIRT1, were highly expressed at 35 dg and their expression levels were significantly higher in the NE35 group than in the RE35 group (Rathbone et al., 2009; Sang-Hyun et al., 2012). Moreover, some genes with critical roles in embryonic development (*PIK3IP1*, *IGFBP2*, and *EGF*) were also downregulated at 35 dg following MCR. The transmembrane protein PIK3IP1 plays a key role in the PI3K/AKT/mTOR signaling pathway, whereas the IGFBP2 and EGF promote DNA synthesis and cell proliferation (Fisher et al., 2005; Song et al., 2015). Interestingly, MCR led to the upregulation of *MSTN*, the major inhibitor of early stage skeletal muscle development (McPherron et al., 1997). These results can explain the molecular basis for the MCR-mediated repression of skeletal myofiber formation during early gestation.

Secondary fibers had formed on the surface of the primary fibers by 55 dg, and histochemical analysis showed that differences in fiber number and size exist between NE and RE groups. Some myogenesis-related genes (*LBX1*, *STMN1*, *IGFBP2*, *PIK3IP1*, and *SPP1*) that were highly expressed at 35 dg, were downregulated at 55 dg. However, the expression levels of some genes coding for myogenic regulatory factors (*MYOG*, *MYF6*, *MEF2A*, and *MEF2C*) peaked at this stage. This was likely due to the continued proliferation and differentiation of myoblasts, as they only undergo terminal differentiation between 65 and 77 dg (Zhao et al., 2011). When myoblasts cease to proliferate, continuation of muscle development involves growth without cell division (Saucedo and Edgar, 2002). Vimentin, the major intermediate filament protein during the early phase of myogenesis, is downregulated as myogenic differentiation proceeds (Cary and Klymkowsky, 1994). We found that *VIM* expression levels were higher in the RE55 group than in the NE55 group. However, the expression levels of *TPM1* and *TPM2*, two genes with roles in myofiber contraction, were downregulated by at the same stage following MCR (Perry, 2001). Moreover, MCR led to the upregulation of the myogenic genes *NFAT5*, *MAML1*, and *SIRT1* at 90 dg. The NFAT5 protein has a combinatorial role in myoblast migration and differentiation, whereas MAML1 functions as a co-activator for MEF2C-mediated transcription and is required for normal myogenesis (Shen et al., 2006; Song et al., 2015). Taken together, these results indicated that significant differences in gene expression patterns exist between primary and secondary fiber formation, and furthered our understanding of the mechanisms underlying the MCR-induced delayed in myotube maturation.

Accumulating evidence has shown that miRNAs have critical roles in the regulation of myogenesis (Chen et al., 2006; Cardinali et al., 2009). In this study, several muscle-specific miRNAs were identified and found to be differentially expressed during prenatal myogenesis. MiR-221 and miR-222 were highly expressed at 35 dg and were subsequently down-regulated at 55 and 90 dg, which agreed well with a previous report indicating that both these miRNAs were downregulated during the transition from proliferation to differentiation (Cardinali et al., 2009). Reductions in the levels of miR-221 and miR-222 are associated with elevated expression of two critical cell cycle inhibitors, p27 and p57 (Cardinali et al., 2009; Liu et al., 2009). The expression of miR-133 peaked at 55 dg, whereas that of miR-1 and miR-206 peaked at 90 dg. These miRNAs act sequentially in the regulation of cell proliferation and myoblast differentiation (Chen et al., 2006; Cardinali et al., 2009). Interestingly, MCR greatly influenced miRNA expression patterns. MiR-106a and miR-221 were downregulated at 35 dg following MCR, whereas miR-1, miR-23a, and miR-206 were downregulated at 55 dg. Although the relevant molecular mechanisms remain unclear, changes of muscle-specific miRNAs involved in myoblast proliferation and differentiation at specific stages is indicative of the existence of novel mechanisms underlying the MCR-mediated repression of myogenesis.

In recently years, numerous algorithms have been developed for miRNA target prediction, and most predictions are based on sequence information and empirically derived rules such as the sequence alignment, sequence conservation between species, and calculation of free energy for miRNA-mRNA complexes (Griffiths-Jones et al., 2008; Friedman et al., 2009; Yue et al., 2009). However, the targets generated by these methods overlap poorly with the small number of validated targets. In this study, we present an integrated approach for adding biological relevance to miRNA target predictions by taking into account the expression of both miRNAs and mRNAs. Moreover, a “miRNA-TF-mRNA” regulatory loop was introduced in this work, which significantly increased the number of biologically relevant miRNA targets. Several novel miRNA-mRNA interactions involved in myogenic signaling pathways were identified, indicating that a complicated and cross-linked regulatory network exists during embryonic myogenesis. Importantly, we further validated the biological functions of several critical regulatory pairs critical for myoblasts differentiation, and found that NC_010454.3_1179 is a novel miRNA that can inhibit myogenesis by downregulating MYOD1. These results not only highlight the importance of using an integrated approach to identify regulatory miRNAs and their targets, but also contribute to our understanding of the molecular mechanisms underlying the repression of myogenesis induced by fetal undernutrition.

In summary, we found that MCR inhibited embryonic myogenesis through the downregulation of critical genes and muscle-specific miRNAs associated with increased cell growth and myoblast differentiation during early gestation in the pigs. An integrative analysis of miRNA and mRNA profiles was used to devise a putative molecular network involved in MCR-mediated regulation of myogenesis. Moreover, we identified NC_010454.3_1179 as a novel miRNA that can suppress myogenesis by downregulating *MYOD1* expression. Our results increased our understanding of how embryonic myogenesis is regulated; the results will also serve as a valuable resource for further investigation of fundamental developmental processes, as well as assist in rational target selection to ameliorate the repression of myogenesis induced by fetal malnutrition.

## Materials and Methods

### Animal Housing and Sample Collection

All procedures involving animals were conducted according to the Regulations for the Administration of Affairs Concerning Experimental Animals (Ministry of Science and Technology, China, revised in June 2004). Forty Large White sows (all sows were selected from the second parity) with an initial body weight of 135.54 ± 3.27 kg were mated with the same boar from the corresponding breed. After mating, the sows were randomly allocated to two groups (*n* = 20) and fed either with a normal diet (3.4 kcal/kg, NE) or an energy-restricted diet (3.0 kcal/kg, RE) diet. There were no discrepancies for the other nutrient components between the two diets (Supplementary Table S2). The daily feed intake was adjusted based on dg, as following: 0–30 dg, 2.0 kg; 31–90 dg, 2.4 kg; and 91–114 dg, 3.0 kg. Sows were housed individually and allowed free access to water. At 35, 55, and 90 dg, four sows from each group were euthanized *via* anesthesia and fetuses were collected. The fetus number and size (e.g., fetus weight and length) were measured. Other sows (eight for each group) were continuously fed till delivering and the reproductive performance (e.g., average number of newborn pigs and average birth weight) were recorded. For histomorphological and sequencing analysis, one fetus (with a fetus weight near to average fetus weight) from each sow was selected (*n* = 4).

### Histochemical Analysis

The *longissimus* muscle was removed from each of the fetuses or neonatal pigs (between the 9th and 10th ribs). A complete cross-section was taken from the muscles and frozen in liquid nitrogen-cooled isopentane. Sections (10 μm) were cut on a microtome (YD-1900, Jinhua YIDI Medical Appliance Co., Jinhua, China) at −20°C and stained with haematoxylin and eosin. Sampling of each section was performed using 12 randomly chosen areas. Each area was photographed at ×200 and ×400 magnifications. The lower magnification pictures (0.7 mm^2^) were used to obtain the mean number of fiber per unit area (density). The higher magnification pictures were used to obtain the mean fiber diameter. Approximately 120 fibers were measured from each section. To explore the distribution of muscle fiber types (slow- or fast-twitch fibers), a conventional histochemical method based on the sensitivity of actomyosin ATPase to acidic pH (pH 4.35) was used (Brooke and Kaiser, 1970).

### Western Blot Analysis

Protein extracts from muscle samples were separated by 10% SDS-PAGE after adding 5 × Laemmli sample buffer and boiling. The separated proteins were transferred onto a PVDF membrane and probed with rabbit polyclonal antibodies against slow-MHC (ab11083, Abcam), fast-MHC (ab91506, Abcam), MYOD1 (ab16148, Abcam), MYOG (ab1835, Abcam), and p21 (ab109199; Abcam). Horseradish peroxidase (HPRP)-conjugated goat anti-rabbit IgG (Santa Cruz Biotechnology) was used as the secondary antibody. For quantification, glyceraldehyde 3-phosphate dehydrogenase (ab8245, Abcam) or tubulin (ab52866, Abcam) was selected as the internal standard. Bound antibodies were detected using the ECL Prime western blotting detection reagent (GE Healthcare) on a CCD-based imager (ImageQuant LAS 4000, GE Healthcare).

### RNA Preparation, Library Construction, and Sequencing

Total RNA was isolated by using Trizol reagent (Invitrogen, Carlsbad, CA, United States) and treated with RQ1 DNase (Promega) to remove DNA. The quality and quantity of the purified RNA were determined by using measuring the absorbance at 260 nm/280 nm (A260/A280) using SmartSpec PLus (Bio-Rad). RNA integrity was further verified by 1.5% agarose gel electrophoresis. PolyA^+^ RNA was purified from 10 μg of the total RNA using Magnetic Oligo (Dt) Beads (Invitrogen). Purified mRNAs were iron-fragmented at 95°C followed by end repair and 5′ adaptor ligation. Reverse transcription (RT) was then performed with RT primers harboring the 3′ adaptor sequence and randomized hexamers in the RNA-Seq Library Preparation Kit for Whole Transcriptome Discovery (Illumina-ompatible). The cDNAs were purified and amplified, and the products corresponding to 200–500 bps were purified, quantified, and stored at −80°C until used for sequencing. For high-throughput sequencing, the libraries were prepared following the manufacturer’s instructions (Illumina, San Diego, CA, United States) and applied to an Illumina Nextseq 500 system for 150-nucleotide (nt) paired-end sequencing by Ablife Inc. (Wuhan, China).

Additionally, 3 μg of total RNA was used for small RNA cDNA library preparation with the Balancer NGS Library Preparation Kit for small/microRNA (GnomeGen) following the manufacturer’s instructions. Briefly, RNAs were sequentially ligated to 3′- and 5′-end adaptors, reverse transcribed to cDNA, and amplified by PCR. The entire library was applied to 10% native PAGE gel electrophoresis and bands corresponding to microRNA insertions were cut and eluted. After ethanol precipitation and washing, the purified small RNA libraries were prepared following the manufacturer’s instructions (Illumina, San Diego, CA, United States) and applied to an Illumina Nextseq 500 system for 150 nt paired-end sequencing by Ablife. Inc. The sequencing data have been submitted to the NCBI Gene Expression Omnibus (GEO) under accession number GSE81751^3^.

### Analysis of Sequencing Data

The raw RNA-seq data were trimmed by removing adaptor tags, low quality tags (with a quality score < 20), and short tags (<16 nt) using the FASTX-Toolkit (version 0.0.13). The clean reads were then mapped to the pig reference genome (Sscrofa10.2) using TopHat2 software (Kim et al., 2013). Based on gene annotation, aligned reads with more than one genome location were discarded. Uniquely localized reads were used to calculate read number and RPKM (Mortazavi et al., 2008). Other statistical results, such as gene coverage and depth and read distribution around start and stop codons, were also obtained. Differentially expressed genes (DEGs) between the test and control samples were analyzed using edgeR (Robinson et al., 2010). The threshold for DEG selection was set as fold change > 2 and *P* < 0.01.

MiRNA analysis was performed using custom Perl scripts and novel miRNAs were predicted by miRDeep2 (v2.0.0.5) (Friedlander et al., 2012). After trimming the 3′ adaptor sequence, all sequences ranging in length from 16 to 26 nt were recorded in a non-redundant file along with copy number. For novel miRNA prediction, the miRDeep2 score cutoff was set to the default value. Differential miRNA expression analysis was performed by Fisher’s exact test. A *P* < 0.01 and fold change > 2 were set as the threshold to define DE miRNAs.

### q-RT-PCR Validation of Differentially Expressed Genes and miRNAs

Total RNA (50 ng) was reverse-transcribed by using Taqman MicroRNA Reverse Transcription Kit (Ambion). Comparative real-time PCR was performed in triplicate using the Taqman Universal PCR Master Mix (Ambion) on the Applied Biosystems 7500 FAST real-time PCR system. Mature miRNA-specific primers and probes were obtained from Riobio Inc. (Riobio Inc., Guangzhou, China). Normalization was performed using RNU6B primers and probes. Relative expression was calculated by the comparative CT method (Livak and Schmittgen, 2001). For real-time PCR analysis of DEGs, total RNAs was reverse-transcribed using a two-step RT-PCR kit (Takara), and expression levels were normalized to that of *GAPDH*. The primers used in this study are shown in Supplementary File S10.

### Functional Enrichment Analysis

Differentially expressed genes were submitted to the DAVID web server^4^ for enrichment analysis (Huang et al., 2009). Enrichment clusters were sorted by the enrichment score in descending order. Categories within clusters were sorted by *P*-value in ascending order. Fold enrichment, Bonferroni and Benjamini corrected *P*-value, and false discovery rate (FDR) were also presented for each category within each cluster. Hierarchical gene set clustering was performed by Cluster3.0. Java TreeView was used to generate a heatmap based on a gene list. Other statistical results were obtained by R software.

### miRNA Target Prediction and Identification of miRNA-mRNA Regulatory Networks

The strategy for identifying miRNA-mRNA regulatory relationships was based on two criteria, namely, computational targets prediction and negative regulation association. As the pig data were not available in TargetScan, miRanda was used for computational target prediction (Enright et al., 2003). We required an alignment score > 145 and energy < -10 kcal/mol, as previously suggested (Xin et al., 2009). Pearson’s correlation coefficients (s (PCCs)) and significant *P*-values were used for identification of causal miRNA-mRNA regulatory relationships (Wang and Li, 2009). The method learns a causal structure from expression data, and applies do-calculus to infer regulatory effects (ranging from −1 to 1). We calculated pairwise causal effects between each DE miRNA and mRNA based on their expression across all samples. The criteria for filtering (selection of a negative regulatory relationship) were set at a correlation coefficient < −0.58 and *P* < 0.05. The filtered miRNA-mRNA pairs with a confident inverse expression pattern were overlapped with the base-pairing target prediction results, yielding the final target prediction result.

### Cell Cultures, *in vitro* Myogenesis Induction and Luciferase Reporter Assays

Myoblasts were isolated through a series of preplating steps as previously described (Rando and Blau, 1994; Geotsch et al., 2015). Briefly, Large White newborn pigs (less than 2 days old) were euthanized and submerged in 70% ethanol for 5 min. The hind limbs were removed and muscle was dissected away from the bone and placed in a few drops of warm phosphate-buffered saline (PBS, pH 7.2). The cells were enzymatically dissociated in 2 mL of a 1 mg/mL collagenase/dispase solution (Roche, Basel, Switzerland). The slurry was incubated at 37°C for 40 min with agitation every 10 min to dislodge the cells. The slurry solution was then filtered through a sterile tea sieve and washed with PBS. The filtrate was then centrifuged at 350 × *g* to pellet the cells. After removing the supernatant, the cell pellet was resuspended in 2 mL primary culture medium. Myoblasts were isolated based on the shorter adhesion time of fibroblasts compared with that of myoblasts. The myoblasts were then cultured and myogenesis was induced as previously described (Lu et al., 2000). For overexpression, the *MYOD1* genes containing the 3′-UTR were cloned into the pcDNA.3.1 vector (Invitrogen, Carlsbad, CA, United States), and transfected into the myoblasts by electroporation methods as described by the manufacturer. The miRNA mimics, control mimics, miRNA inhibitor, and control inhibitor were purchased from Riobio Inc. and transfected into cells using Lipofectamine 2000 (Invitrogen).

To create 3′-UTR luciferase reporter constructs, fragments of 3′-UTRs of selected target genes were cloned downstream of a CMV-driven firefly luciferase cassette in the pMir-REPORT vector (Ambion). For miRNA target validation assays, 1 × 10^5^ HEK293 cells in a 24-well plate were transiently transfected with 10 ng of each firefly luciferase reporter plasmids, 10 ng of the pCSK-lacZ vector (for normalization), and 150 ng of miRNA mimics or inhibitors using Lipofectamine 2000 (Invitrogen) according to the manufacturer’s protocol. The transfection experiment was performed in triplicate. After 48 h, cells were lysed and the luciferase activity was determined using a GoMax 96 Microplate Luminometer (Promega). Firefly luciferase activity was normalized to that of β-galactosidase.

## Data Availability Statement

The datasets generated for this study can be found in the sequencing data have been submitted to the NCBI Gene Expression Omnibus (GEO) under accession number GSE81751 (http://www.ncbi.nlm.nih.gov/geo).

## Ethics Statement

The animal study was reviewed and approved by the Regulations for the Administration of Affairs Concerning Experimental Animals (Ministry of Science and Technology, China, revised in June 2004).

## Author Contributions

YH, BY, and XW performed the histochemical and biochemical analyses. JH and XW analyzed the sequencing data and helped to draft the manuscript. DC and BY conceived and organized the study. All authors contributed to the article and approved the submitted version.

## Conflict of Interest

XW was employed by the company of ABlife Inc. The remaining authors declare that the research was conducted in the absence of any commercial or financial relationships that could be construed as a potential conflict of interest.

## Footnotes

1 https://david.ncifcrf.gov/

2 http://bioinfo.life.hust.edu.cn/AnimalTFDB/

3 http://www.ncbi.nlm.nih.gov/geo

4 http://david.abcc.ncifcrf.gov/

## Supplementary Material

FIGURE S1 Morphology of the *longissimus* muscles using haematoxylin and eosin staining. All areas were photographed at a magnification of ×400; NE/RE indicate normal/reduced calorie supply during gestation; E35, E55, and E90 indicate samples collected at 35, 55, and 90 days of gestation (dg), and P01 indicate 1 day post-partum.

FIGURE S2 Heatmap shows the expression pattern of all DE miRNAs identified during the prenatal muscle development. The sequencing data (reads) were normalized by tags per million (TPM) values [TPM = miRNA total reads/total clean reads × 10^6^].

TABLE S1 Effect of maternal calorie intake on fetal development and reproductive performance of sows (*n* = 20/diet).

TABLE S2 Ingredient and chemical composition of the experimental diets.

FILE S1 RNA/miRNA sequencing data mapping and annotation.

FILE S2 Functional clustering analysis of DE genes at different stages. Top ten significant clusters from each set were selected based on their enrichment scores.

FILE S3 Details of the DE genes identified between NE and RE group at different stages.

FILE S4 Q-PCR validation of RNA/miRNA sequencing data. The results are presented by fold changes of transcript abundance in muscle samples. For the NE35 sample, the fold change in gene/miRNA expression relative to the NE35 equals one, by definition. For each row, different superscript letters indicate a statistically significant difference (*P* < 0.05) in gene expression (Q-PCR) among different groups.

FILE S5 Details of the DE miRNAs identified between NE and RE group at different stages.

FILE S6 Complete lists of DE miRNAs for various comparisons in Venn diagrams.

FILE S7 miRNA-mRNA regulatory pairs and critical signaling pathways identified during the prenatal muscle development.

FILE S8 miRNA-TF interactions identified during the prenatal muscle development.

FILE S9 Lists of the TF-mRNA interactions.

FILE S10 Lists of the primers used in this study.
