# Corrigendum: Transcriptome Characterization of Repressed Embryonic Myogenesis Due to Maternal Calorie Restriction

**DOI:** 10.3389/fcell.2020.00667

**Published:** 2020-07-29

**Authors:** Jun He, Ying He, Bing Yu, Xuelian Wang, Daiwen Chen

**Affiliations:** ^1^Institute of Animal Nutrition, Sichuan Agricultural University, Chengdu, China; ^2^Key Laboratory of Animal Disease-Resistance Nutrition, Ministry of Education, Chengdu, China; ^3^ABlife Inc., Wuhan, China

**Keywords:** embryonic myogenesis, development, transcriptome, myofiber, nutrition

An author name was incorrectly spelled as Xulian Wang. The correct spelling is Xuelian Wang.

The authors apologize for this error and state that this does not change the scientific conclusions of the article in any way. The original article has been updated.

