# Supplementary figures and images for: Transcriptome Characterization of Repressed Embryonic Myogenesis Due to Maternal Calorie Restriction
Source: Front Cell Dev Biol. 2020 Jun 26;8:527. doi: 10.3389/fcell.2020.00527 (PMC7332729; doi:10.3389/fcell.2020.00527)

**E35**

**E55**

**E90**

**P01**

**NE**

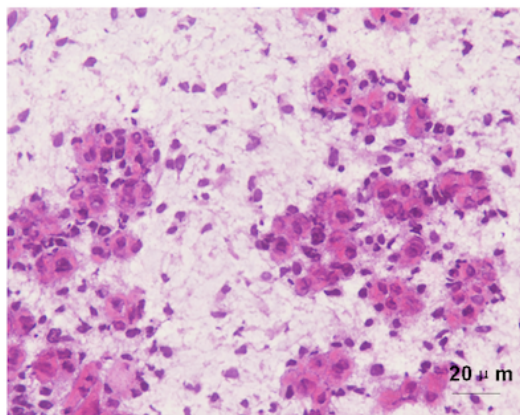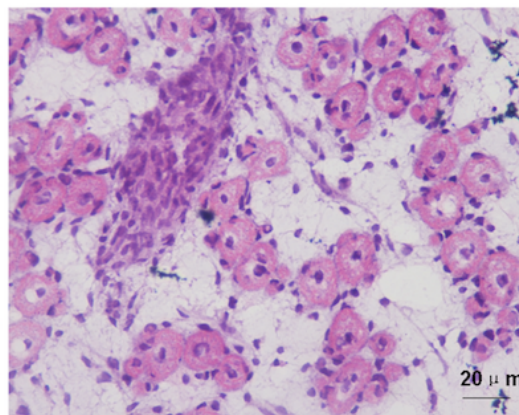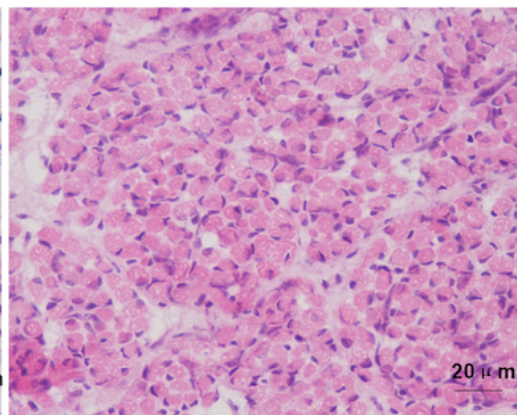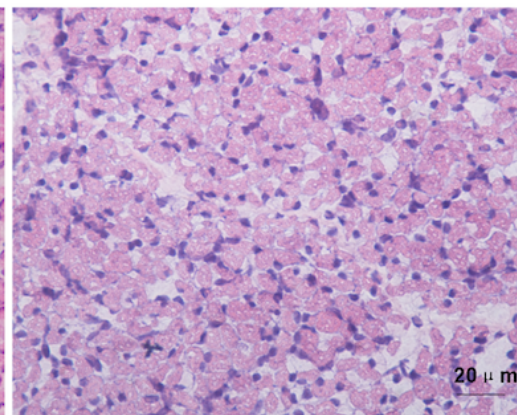

**RE**

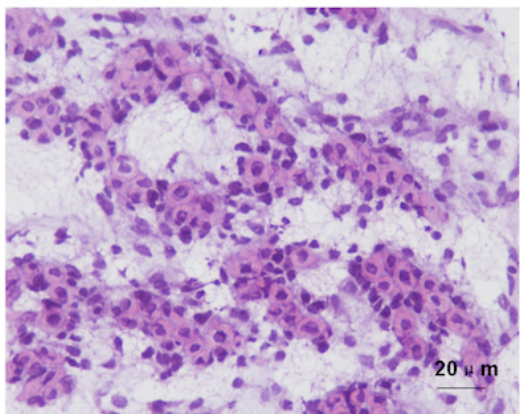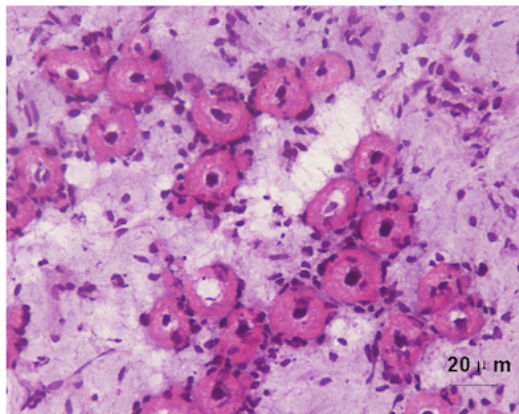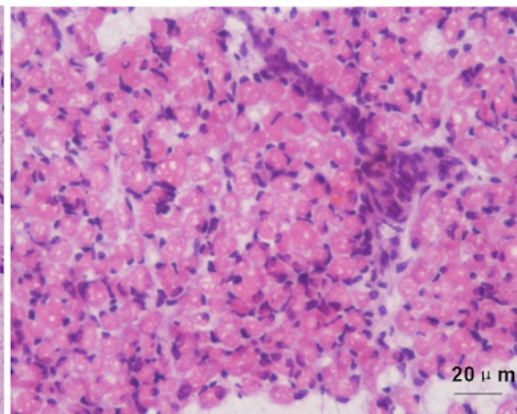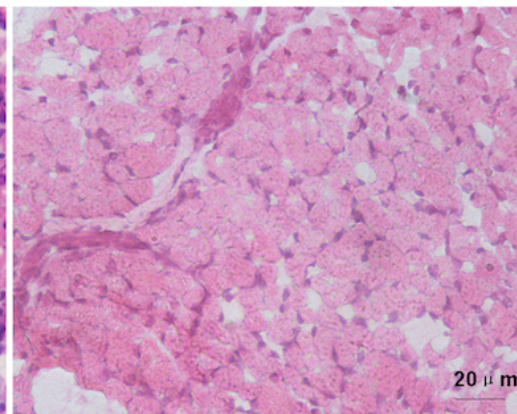

Supplement: FIGURE S1 — Morphology of the longissimus muscles using haematoxylin and eosin staining. All areas were photographed at a magnification of ×400; NE/RE indicate normal/reduced calorie supply during gestation; E35, E55, and E90 indicate samples collected at 35, 55, and 90 days of gestation (dg), and P01 indicate 1 day post-partum. [file Data_Sheet_11.PDF]

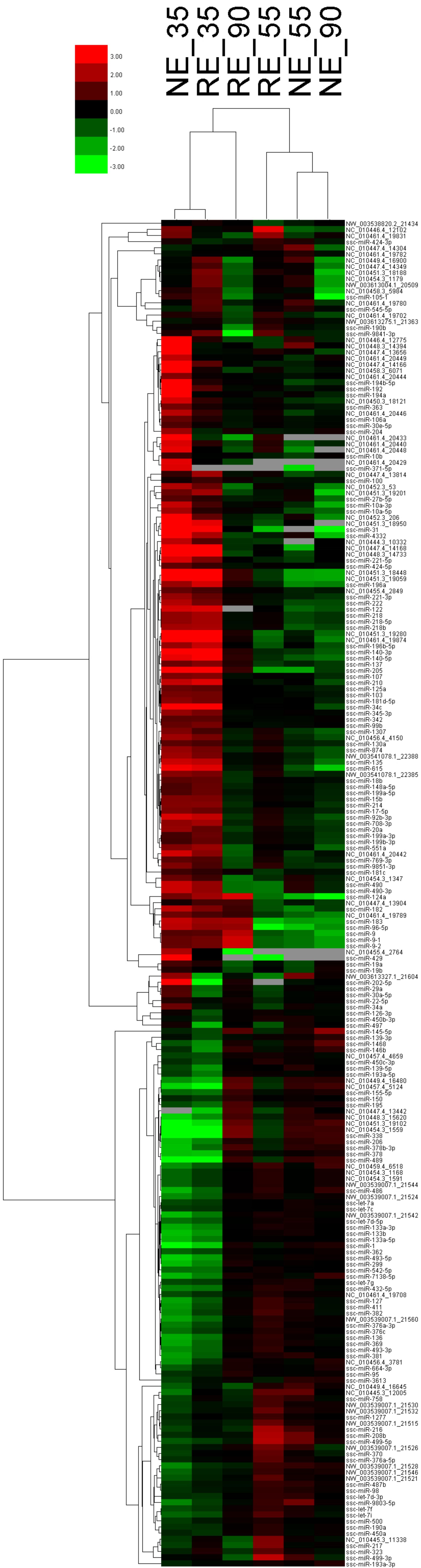

Supplement: FIGURE S2 — Heatmap shows the expression pattern of all DE miRNAs identified during the prenatal muscle development. The sequencing data (reads) were normalized by tags per million (TPM) values [TPM = miRNA total reads/total clean reads × 106]. [file Data_Sheet_12.PDF]
