# Supplementary material for: Transcriptome Characterization of Repressed Embryonic Myogenesis Due to Maternal Calorie Restriction
Source: Front Cell Dev Biol. 2020 Jun 26;8:527. doi: 10.3389/fcell.2020.00527 (PMC7332729; doi:10.3389/fcell.2020.00527)
Supplement: TABLE S1 — Effect of maternal calorie intake on fetal development and reproductive performance of sows (n = 20/diet). [file Table_1.DOC]

**TABLE S1: Effect of maternal calorie intake on fetal development and reproductive performance of sows (n=20/diet).**

| **Stages** | **Energy levels** | **Average total no. fetuses / sow** | **Average no. living fetuses / sow** | **Fetal weight (g)** | **Fetal length (cm)** | **Average total no. born / litter** | **Birth weight (kg)** |
| --- | --- | --- | --- | --- | --- | --- | --- |
| **E35** | **NE** | 15.25±0.95 | 15.00±1.08 | 5.28±0.17 | 3.72±0.10 | - | - |
|  | **RE** | 14.75±1.03 | 13.25±1.31 | 6.00±0.39 | 4.00±0.10 | - | - |
| **E55** | **NE** | 12.75±0.95 | 13.67±0.33 | 92.59±2.21 | 12.15±0.18 | - | - |
|  | **RE** | 14.50±0.96 | 14.25±1.18 | 86.07±3.56 | 11.85±0.28 | - | - |
| **E90** | **NE** | 12.50±0.29 | 12.25±0.48 | 780.03±11.81 | 24.53±0.18 | - | - |
|  | **RE** | 14.67±0.88 | 14.33±0.67 | 647.18±31.12^**^ | 23.46±0.33^*^ | - | - |
| **P0** | **NE** | - | - | - | - | 12.00±0.82 | 1.58±0.13 |
|  | **RE** | - | - | - | - | 12.25±0.28 | 1.33±0.21^*^ |

Note: E35, E55, E90 means samples were collected at 35, 55, and 90 days of gestation (dg); P0 means the first day of delivering. Sows were fed with a normal (NE) or calorie-restricted (RE) diet, and details of the sample collection were provided in the text. All data were expressed as “Mean±SEM”. ^*^Means *P*<0.05; ^**^Means *P*<0.01.
