# Supplementary material for: Transcriptome Characterization of Repressed Embryonic Myogenesis Due to Maternal Calorie Restriction
Source: Front Cell Dev Biol. 2020 Jun 26;8:527. doi: 10.3389/fcell.2020.00527 (PMC7332729; doi:10.3389/fcell.2020.00527)
Supplement: TABLE S2 — Ingredient and chemical composition of the experimental diets. [file Table_2.DOC]

**TABLE S2.** **Ingredient and chemical composition of the experimental diets**

| items | NE | RE |
| --- | --- | --- |
| *Ingredient, %* | | |
| corn | 45 | 45 |
| Soybean meal | 13.6 | 13.6 |
| Wheat bran | 27.8 | 27.8 |
| Soybean oil | 9.1 | 4.5 |
| Wheat fiber | 0 | 2.54 |
| Soybean fiber | 0 | 1.1 |
| Zein fiber | 0 | 0.96 |
| salt | 0.4 | 0.4 |
| choline | 0.14 | 0.14 |
| Calcium carbonate | 1.24 | 1.24 |
| Calcium bicarbonate | 1.99 | 1.99 |
| lysine | 0.1 | 0.1 |
| threonine | 0.1 | 0.1 |
| premix^1^ | 0.53 | 0.53 |
| total | 100 | 100 |
| *Chemical composition, %* | | |
| DE (Mcal/Kg) | 3.40 | 3.00 |
| CP (%) | 13.49 | 13.92 |
| EE (%) | 2.95 | 2.95 |
| Lys (%) | 0.69 | 0.69 |
| SLD Lys (%) | 0.60 | 0.60 |
| Met+Cys (%) | 0.35 | 0.35 |
| Thr (%) | 0.46 | 0.46 |
| Ca (%) | 0.96 | 0.96 |
| TP (%) | 0.79 | 0.79 |
| AP (%) | 0.48 | 0.48 |

Note 1: Provided the following (per kilogram of complete diet): 170 mg of Fe; 17 mg of Cu; 160 mg of Zn; 35 mg of Mn; 0.3 mg of Se; 0.28 mg of I; 15,500 IU of vitamin A; 3,250 IU of vitamin D3; 16 IU of vitamin E; 5.2 mg of riboflavin; 20 mg of nicotinic acid; 11 mg of pantothenic acid; 0.12 mg of vitamin B12; 0.13 mg of biotin.
